# Nurse Leaders' Perceptions of Followership Development Needs: A Descriptive Qualitative Study

**DOI:** 10.1155/jonm/7920607

**Published:** 2025-04-07

**Authors:** Welile M. Mamba, Willem Fourie, Tanya Heyns

**Affiliations:** ^1^Department of Nursing Science, University of Pretoria, Pretoria, South Africa; ^2^School for Data Science and Computational Thinking, Stellenbosch University, Stellenbosch, South Africa

**Keywords:** development, followership, needs, nurse leaders, nurses

## Abstract

**Introduction:** Followership is a critical aspect of leadership because, without followers, there can be no leaders. To be successful, organizations must develop high-performance, self-developed, and self-led followers with specific values. However, organizations have traditionally prioritized developing leaders, leaving out followers in the development equation. Followership development allows nurses to learn how to work effectively in teams, fostering teamwork skills.

**Aim:** To explore nurse leaders' perceptions of followership development needs.

**Materials and Methods:** A descriptive qualitative design was used. Data were collected via face-to-face, semistructured, in-depth interviews with 10 purposively selected nurse leaders (middle and top management). The interviews were audio-recorded, transcribed verbatim, and analyzed via Braun and Clarke's approach.

**Findings:** Participants emphasized the need for followership education for nurses. Followership should be incorporated into undergraduate nursing curricula and in-service training. Additionally, participants verbalized the need for a followership development program that should train nurse followers in communication, interprofessional collaboration, decision-making, team building, teamwork, and leadership. The participants suggested that followership could be developed by providing incentives for good followership behavior and conducting periodic performance appraisals.

**Conclusion:** The participants emphasized the importance of a followership development program, which may enhance the relationship between nurse leaders and followers and contribute to positive patient outcomes. Future research needs to develop programs to develop nurses who are followers.

**Implications for Nursing Management:** Developing followers in a hospital setting is critical. Understanding needs and what a followership program should include assists nurse leaders in planning and implementing relevant programs.

## 1. Introduction

Leadership does not occur in isolation since leaders work with followers, and followership is responsible for many successful organizational outcomes [1]. Leaders and followers have an inextricable and symbiotic relationship [2]. They are bound by a complex emotional web and perform distinct but interdependent activities [3]. Followership is a critical aspect of leadership because, without followers, there can be no leaders [4, 5]. The partnership between followers and leaders is essential for personal and organizational success [6]. Hence, through shared leadership, leaders and followers need to have a collective sense of responsibility and commitment to achieving the vision and mission of an organization [6]. Cooperation between followers and leaders to accomplish tasks and improve communication and problem-solving capabilities through various cognitive and behavioral mechanisms is vital for developing trusting relationships and enhancing team performance [3]. Moreover, leadership is a process that develops over time and depends on the characteristics of leaders and followers [7].

Followership describes how individuals respond to and interact with their leaders [8, 9]. Followership is broadly defined via two lenses. First, followers do not formally occupy leadership positions in organizations, and second, being a follower is a socially constructed process [1]. Followers adopt goals and willingly accept the influence of their leaders [10]. Thus, “following” is a human response to a leader's behavior and perceived power in relation to individual beliefs. Therefore, accepting a follower role is based on an individual's motivational response to leader-initiated stimuli, with the follower's action aligned with his or her perception of benefit and his or her attitudinal response to the leader's action [11]. Followership does not imply an individual deficiency but rather a different role in the leadership process [12]. Effective followership depends on the organizational structure, personal characteristics, and follower beliefs [12].

The word “follower” may refer to a personality type, a hierarchical position, a role, traits, or behaviors. Although followers do not have authority, they have power and influence [8]. The influence of followers on leaders and other team members should be considered [4]. Followers exert influence to execute their leaders' vision or accomplish organizational goals [6]. Through their mindfulness, purposely paying attention, and remaining nonjudgmental, followers contribute to team cohesion [3].

Shared leadership, also known as distributed leadership, is important in followership because leadership is performed collectively by leaders and followers but at different times [13]. By allowing followers to function as leaders within the team, shared leadership changes the role of followers from passive to active such that they can be described as effective followers characterized by a sense of personal responsibility for achieving the defined goals, sharing the organizational mission [14], and improving teamwork [15]. Nurse leaders should adopt relational leadership styles to create safe and healthy organizational cultures that support followers in providing patient-centered, high-quality, and cost-effective care. Relational leadership styles among nurse leaders improve followers' well-being and work engagement and enhance positive relationships and team collaboration [16].

Organizations have traditionally prioritized developing leaders, leaving out followers in the development equation [17]. Successful organizations must develop high-performance, self-developed, and self-led followers with specific values [17]. Follower development involves creatively exploring new possibilities in the organizational process. Moreover, follower development prepares individuals to seamlessly transition to effective leadership [6, 17]. Hence, in hospitals, followership development allows nurses to learn how to work effectively in teams, fostering teamwork skills [9]. Although followership development is essential in nursing [18], there is a paucity of research on followership development needs for nurses. Therefore, this study explored nurse leaders' perceptions of followership development needs in hospital settings. Exploring nurse leaders' perceptions may promote “follower” inclusivity in hospitals [19] because leaders can be barriers to or facilitators of effective followership [18]. Exploring needs before developing a program allows interventions to be designed that meet the specific needs of nurses and avoid wasted resources [20]. Hence, this study will inform the development of a followership development program for nurses working in hospitals.

## 2. Materials and Methods

### 2.1. Research Question

The central question in this study was “What are nurse leaders' perceptions of followership development needs?”

### 2.2. Study Design

This descriptive qualitative study aimed to generate in-depth data about a poorly understood phenomenon to provide healthcare solutions [21], namely, nurse leaders' perceptions of followership development needs. The Consolidated Criteria for Reporting Qualitative Research (COREQ) checklist was used to report this study [22] (supporting information available (here)).

### 2.3. Research Setting

This study was conducted at a public referral hospital in the Kingdom of Eswatini. The hospital has a total bed occupancy of 500 patients, and approximately 700 outpatients are treated daily. In the hospital, nurse leaders include middle and top management, whereas followers include all registered nurses who do not occupy leadership positions. The setting, which functions as a teaching hospital for the university where the lead author works, was conveniently chosen.

### 2.4. Sampling Method and Procedure

Purposive sampling was used to select participants who were aware of followership development needs, as suggested by Doyle et al. [23]. As nurse leaders, the participants are currently the followers of their superiors. Additionally, before becoming leaders, the participants had experience in follower roles. Hence, their perceptions reflect their awareness of followership development needs. Nurse leaders in middle and top management positions who were working full time for at least 40 h per week and had been in a nurse leadership position for 6 months or more were included in the sample. After being granted permission by the hospital to conduct the study, the lead author recruited the participants by visiting each unit to explain the study. The participants who met the inclusion criteria and were willing to participate were given an information leaflet. Dates and times were also agreed upon to clarify questions, sign informed consent, and schedule interview times and places.

### 2.5. Data Collection and Management

The lead author, a male university lecturer with a postgraduate degree and clinical experience in critical care nursing, visited willing participants in their offices. During this visit, the questions were clarified, and an informed consent form was signed. Two pilot face-to-face interviews with participants in the selected hospital were conducted via an unstructured interview guide developed by the research team. The pilot interviews helped revise and refine the questions and minimized problems that may have arisen during data collection [24]. The interview guide was not changed; therefore, the data collected were included in the analysis. The question posed was as follows: What should be included in a program to develop followers in this hospital? The pilot interviews were audio-recorded with participant consent.

Following the pilot interviews, the lead author conducted eight additional face-to-face interviews. The interviews were conducted at convenient times and venues to avoid patient care disruptions. Data saturation was reached after the ninth interview, and an additional interview was conducted to verify saturation. Verbatim transcripts were sent to the research team for validation. The second author is a male university lecturer with teaching and research experience in Management Sciences. The third author is a female university lecturer with education and clinical experience in emergency and critical care nursing. The participants did not feel coerced to participate because all the authors were outsiders and had no supervisory relationships with the participants.

### 2.6. Data Analysis

The audio-recorded interviews were transcribed verbatim via Microsoft Office Word 365. The data were analyzed using thematic analysis following the Braun and Clarke [25] approach. The lead author familiarized himself with the data by reading and rereading the transcripts, listening to the recordings, and writing down word-for-word what was captured in the audio recordings. The lead author then generated initial codes using short phrases. Similar codes were then sorted into categories. The lead author then searched for themes by identifying shared subthemes in the participants' responses. The themes and subthemes were reviewed and refined in collaboration with the research team, and a consensus was reached on the final themes and subthemes.

### 2.7. Trustworthiness

Credibility, dependability, transferability, and confirmability are used to increase trustworthiness [26] (Table 1).

### 2.8. Ethical Considerations

This study followed the Declaration of Helsinki [27]. Ethical approval was granted by the Faculty of Health Sciences Research Ethics Committee, University of Pretoria (662/2022), and the Eswatini Health and Human Research Review Board (EHHRRB028/2023). The hospital administration granted permission to conduct the study. All participants received oral and written information about the study. Written consent to participate was obtained from all participants. The participants were informed about their right to withdraw their informed consent at any time without retribution. They were assured that pseudonyms would be used in the research report to maintain confidentiality. Furthermore, the research team did not envision emotional or psychological harm arising from the interviews.

## 3. Findings

### 3.1. Characteristics of Participants

The participants included 10 female nurse leaders, two from top management and eight from middle management, between December 2023 and March 2024. Nine participants had a bachelor's degree, and one had a master's degree. The mean age of the participants was 54 years (±3.75 standard deviation [SD]), and the mean leadership experience was 11 years (±2.91 SD). Each interview lasted between 30 and 45 min.

### 3.2. Thematic Analysis

Two themes and five associated subthemes emerged from the data (Table 2).

#### 3.2.1. Theme 1: Followership Education

The participants expressed a need to incorporate followership into undergraduate nursing curricula and conduct in-service education for nurses. Additionally, the participants voiced a need for a followership training program for nurses in the hospital.

##### 3.2.1.1. Subtheme 1: Incorporation of Followership Into Nursing Education Curricula

The participants expressed the need for followership to be included in undergraduate nursing curricula, as this would allow nursing students to learn about and appreciate followership in the earliest stages of their careers. Moreover, incorporating followership into curricula will foster effective followership skills and enhance team performance among students:

*“…The [undergraduate] student nurses must be trained so that they come out already knowing about followership and how to follow leaders so that they grow with it in the profession…”* (Participant 10).

*“Actually, this followership should start at tertiary when they [undergraduate nursing students] are being trained… I think that there must be a course concerning how to be a good follower or best follower…”* (Participant 9).

The participants further suggested that hospitals and nurse-training schools collaborate on followership education. This collaboration will facilitate learning on followership for nursing students and enhance the acquisition of knowledge on followership for nurses who are already working:

*“…include collaboration with the tertiary institutions. I'm thinking that if we can collaborate with them because some of us were trained a long time ago, it can help us to be on board [concerning followership]. The collaboration will assist because they do send their students for attachment in the hospital, so there will be a good follow-up of the students to see if what they have learned in class [on followership] blends well with what is in the clinical area”* (Participant 1).

##### 3.2.1.2. Subtheme 2: In-Service Education on Effective Followership

The participants revealed the need for currently employed nurses to receive in-service education on followership. In-service training will enhance the understanding of followership and promote effective and exemplary followership among nurses:

*“…in the working environment, as leaders, we need to organize some in-service trainings for our staff—to invite somebody to teach them on how to be good followers…”* (Participant 7).

*“…nurses who are already working must be trained on followership maybe through in-service trainings. Maybe some would even notice the importance of followership…it would be very helpful…”* (Participant 9).

These responses reflect that nurses understand the importance of followership in hospital settings.

##### 3.2.1.3. Subtheme 3: Followership Training Program and Proposed Content

The participants expressed a desire for a formal followership training program for nurses in the hospital. According to the participants, the followership program must focus on training nurses to follow leaders. The training program should train nurses on the characteristics of a good follower and how followers need to interact with leaders in a hospital setting. The professional growth and development of nurses will also be enhanced:

*“I think there must be a program—a training program just for followers. Something not related to work. Something not related to the company—just a program that is just for the training of nurses for their growth and development”* (Participant 3).

*“I wish there could be training for followership, especially in this young generation. I think they need to be given something written in black and white because some of them do not know how to follow… I feel like the training is needed on how to be a follower—the characteristics that you need to have as a follower and how one needs to follow a leader—the formal training is required”* (Participant 10).

Moreover, the participants described the content that should be included in the followership training program. Firstly, participants voiced the need for training followers on communication skills. Training followers on communication skills is essential to capacitate them to respectfully challenge their leaders. Additionally, followers will be equipped with positive communication skills, which include active listening when interacting with leaders and other followers. Positive communication between nurse leaders and followers is important to prevent medical errors:

*“…Communication skills. They should be able to challenge even the leader if there are any problems in the ward [unit]… followers can be intimidated - maybe the leader can intimidate them, but they should be able to feel free to challenge the authority. Not that the decision of somebody who is in authority in the unit cannot be challenged… it revolves around the communication. It is one of the components that can be included because that is now a characteristic that would be taught to the followers to be able to challenge the leader but with respect…”* (Participant 4).

*“They must be taught on communication skills. Communication skills are very important. Positive communication is very important, as are listening skills”* (Participant 10).

The participants further expressed that the program should also include training followers on leadership skills. The intention is to enable followers to function in leadership positions should an opportunity arise when a leader is temporarily not available due to reasons such as being on leave of absence. Moreover, the program needs to incorporate mentoring of followers by nurse leaders. The aim is to equip followers with knowledge and skills that they would need when promoted to leadership positions in the future:

*“…competences in leading others, such as emotional intelligence, coaching skills, being self-motivated, and being willing to learn. They should be taught about being open-minded and being flexible…”* (Participant 8).

*“I think as followers, we need to be trained on leadership skills. I think the leadership skills are very important because one day you will be promoted to be a leader…”* (Participant 7).

“*The program should also include mentoring of followers by leaders. We have to mentor those nurses… You are just preparing them for their leadership skills - maybe they will be promoted one day. They have to know what is happening on that side”* (Participant 3).

Another component that should be included in the training program is interprofessional collaboration. The participants expressed that the followers need to be taught about interprofessional collaboration to enable them to work harmoniously and effectively with other members of the healthcare team to improve patient outcomes. Additionally, training on interprofessional collaboration will alleviate conflict among followers and other members of the healthcare team. The reason is that conflict compromises patient care:

*“…The program must include interprofessional collaboration with other people so that nurses are able to work with other healthcare professionals as a team… what I have seen in my unit is that… there's friction. It means something is just lacking… and it compromises the patient's care”* (Participant 3).

The participants voiced that the followership program must include capacitating nurses with decision-making skills. Followers will be able to contribute to shared decision-making in the hospital rather than relying on the leader to make decisions. Follower involvement in shared-decision-making may improve their work performance and job satisfaction:

*“Followers need to also be taught on decision-making. It cannot be that all decisions are made by the leader in the unit [laughs]. Most of the time, what the leader needs to make decisions comes from the followers”* (Participant 10).

Participants also voiced that the followership program needs to train nurses on team building and teamwork. Team building enhances the psychological well-being of nurses:

*“The program can also include team building… I don't know how I can put this, but the program can help in that there are times where you need to distress so that you can perform better at work”* (Participant 1).

Moreover, training followers on teamwork will promote collaboration and harmonious working relationships among followers and leaders. Patient outcomes will be improved:

*“Teamwork at times was not there among the followers. it is always a challenge. I do not know why. You do not have the same goals, so to say. Therefore, this program must include the teamwork component so that nurses can learn to work as a team”* (Participant 7).

#### 3.2.2. Theme 2: Followership Development Through Recognition of Followers

This theme describes the need for followership development through the recognition of followers. Participants expressed the need for developing followers through conducting periodic performance appraisals and providing incentives for good followership behavior among nurses.

##### 3.2.2.1. Subtheme 1: Conducting Performance Appraisals

Participants expressed the need to conduct quarterly performance appraisals. Conducting performance appraisals is an approach that will enhance good followership behaviors among nurses:

*“…performance appraisals to be conducted quarterly…These performance appraisals can be conducted quarterly so that they can encourage the followers to do the right thing every day”* (Participant 7).

##### 3.2.2.2. Subtheme 2: Rewarding Good Followers

The participants voiced the need for developing followers through rewarding them with incentives and tokens for demonstrating good followership behaviors. Incentives and tokens are a way of motivating followers to be role models of good followership to other nurses. Positive reinforcement through rewards will boost the morale of followers and promote a healthy working environment:

*“Maybe we can give them—I think it is sort of encouraging—a token if there is the best follower in our units…smaller goodies to encourage them in our units”* (Participant 7).

*“…apart from formal training, I think there should be some sort of incentive for the one who is a good follower. I think they must be given something to encourage them so that they teach others and be an example to others on how to be good followers. It would also make them happy that they are working in an environment where good followership is recognized…”* (Participant 10).

## 4. Discussion

This study explored nurse leaders' perceptions of followership development needs for nurses working in hospital settings. The participants expressed a desire for a followership program for nurses. A followership development program is an innovative approach that requires an inclusive organizational culture that is sensitive to “bottom-up” changes as opposed to the usual “top-down” approach [28]. Since followership programs foster followership within an organization [28], leaders should be responsible for developing followers [29]. The development of a followership program will facilitate role clarification for nurse followers concerning their contribution to the leader−follower relationship in the hospital. Moreover, follower role clarification improves teamwork and patient care [30]. A lack of follower role clarification compromises the quality of patient care, the work environment, and the productivity of nurses [30]. In this study, we asked leaders whether there was a need for a nurse followership program and what they thought such a program should look like.

The participants suggested that undergraduate nursing curricula should include followership training. Incorporating followership concepts into nursing education and continuous development training programs will improve nurses' knowledge and understanding of followership and its importance for clinical practice and patient safety [18]. The inclusion of followership in undergraduate nursing programs enhances the smooth transition of nursing students into the workplace and contributes to a competent and dynamic workforce [30]. Followership education includes learning the fundamentals of followership and learning what constitutes a follower who is ethical and effective. Additionally, the curriculum should include the principles of good followership along with those of good leadership [8]. The following topics may also be included in followership classes: followership theory and practice, followership in groups and communities, and enacting change as a follower [31]. Followership training also involves knowledge, beliefs, attitudes, behaviors, and skills that can improve team effectiveness [32]. Thus, enabling nursing students with followership knowledge will help them understand how to collaborate with leaders to improve patient outcomes and organizational success. The participants further highlighted the importance of conducting in-service followership training. Not all nurse followers should be assumed to have followership skills, which include self-management, responsibility, integrity, commitment, competence, focus, ownership, and versatility, as these skills must be learned and practiced before they are mastered [30]. Hence, in-service training, coaching, and mentoring facilitate the development of followership skills [28].

Our study participants suggested that nurse followers receive incentives to demonstrate good followership behaviors as a form of motivation. Appropriate reward systems can build good followership into the organizational fabric. Additionally, reward systems indicate that organizations recognize the importance of followership [3]. Therefore, giving rewards for good followership may enhance performance and commitment. Other nurse followers will be motivated to adopt positive followership behaviors, resulting in improved patient care and outcomes. The participants also expressed a need to increase the frequency of performance appraisals. Performance appraisals may be conducted to measure and provide feedback on followership skills [28].

According to the participants, nurse followers must be trained in communication skills. This finding aligns with that of Chukwuma [17], where participants highlighted that communication is a critical skill for effective followers. Communication should be direct, purposeful, and not hesitant during critical moments [33]. Communication between leaders and followers enhances work relationships, productivity, and the quality of patient care [30], whereas communication breakdown can lead to medical errors and adverse hospital events [34]. In our study, participants noted that nurse followers need to be taught leadership skills so that they are ready to be future leaders in hospital settings. Chukwuma [17] reported that followers are seldom equipped to be future leaders, especially if organizations have a counterproductive, myopic view of followership. Additionally, in our context, nurse leaders are promoted to their positions on the basis of years of service without undergoing training to acquire leadership qualifications from a recognized university.

The participants also suggested that nurse followers be trained in interprofessional collaboration. Effective followership in interprofessional healthcare teams may reduce medical errors, leading to improved patient outcomes and more efficient utilization of resources [35–37]. During interprofessional collaboration, nurse leaders and nurse followers switch their roles as appropriate to reach their goals. For example, a nurse follower may be required to take a leadership role where expertise is needed to direct patient care [37]. Consequently, the participants also voiced that nurse followers need to receive training in decision-making. Similarly, Chukwuma [17] reported that failure to empower and decentralize decision-making to followers hinders followership development. Allowing followers to actively participate in decision-making and engaging leaders in attaining organizational goals ensures that they are not confined to subordinate positions [17]. Nurse followers should also be trained in team building and teamwork. Team building is the act of meaningfully connecting team members to improve overall group performance and morale [38]. Team building is a common strategy for improving performance and helping followers and leaders learn how to work together [39]. Poor team building results in disillusionment, low morale, and negative motivation [40]. Teamwork is vital for developing followers and harnessing their talent to increase organizational productivity [39]. Poor teamwork has been shown to increase the incidence of missed nursing care, which has been used as an indicator of quality and safety [41].

## 5. Conclusion

The study revealed that there is a need for the development of nurse followers through followership education. To enhance followership development, participants suggested that followership education must be included in undergraduate nursing curricula and that in-service training must be conducted with nurses regarding followership. Moreover, a followership development program that should train nurse followers in communication, interprofessional collaboration, team building, teamwork, decision-making, and leadership needs to be implemented. Designing and implementing a followership development program may clarify the roles of nurse followers and their contribution to healthcare teams. Moreover, a followership program may improve the relationship and collaboration between nurse leaders and followers, resulting in positive patient outcomes.

### 5.1. Implications of the Study

This study highlights the need for followership development programs for nurses working in hospitals. Nurse leaders need to view followership as a skill that can be developed through training. The formal development of followers may result in improved healthcare team partnerships and reduced medical errors.

### 5.2. Strengths and Limitations

One strength is that this study provides insight into nurse leaders' perceptions of followership development needs in hospital settings. This study has several limitations. The study explored only the perceptions of nurse leaders and excluded nurse followers and other healthcare professionals. The study's findings are contextual and cannot be generalized because the study adopted a qualitative research approach and was conducted only in a single hospital.

### 5.3. Recommendations for Further Research

Further research needs to develop a followership program for nurses working in hospital settings.

## Figures and Tables

**Table 1:** Summary of strategies implemented to enhance trustworthiness.

Criteria	Activities
Credibility	Prolonged engagement occurred between the interviewer and participants before and during interviews. Member checking occurred when participants were asked to verify the transcripts and if the findings reflected their perceptions
Dependability	An audit trail of the data collection, management, and analysis was kept
Transferability	Purposive sampling of nurse leaders and providing a thick description of the participants, research setting, and methodology
Confirmability	Adequate and relevant direct verbal quotations from the participants were used to support the findings

**Table 2:** Summary of themes and subthemes.

Theme	Subthemes
Followership education	Incorporation of followership into nursing education curricula
	In-service education on effective followership
	Followership training program and proposed content

Followership development through recognition of followers	Conducting performance appraisals
	Rewarding good followers

## Data Availability

The data that support the findings of this study are available upon request from the corresponding author. The data are not publicly available due to privacy or ethical restrictions.

## Supporting Information

The Consolidated Criteria for Reporting Qualitative Research (COREQ) checklist included as supporting information guided the reporting of the study. The checklist has different components that researchers must follow when reporting qualitative studies (see supporting file: ISSM_COREQ_Checklist for clarity).
